# Gender differences in HIV knowledge among adolescents and young people in low-and middle-income countries: a systematic review

**DOI:** 10.3389/frph.2023.1154395

**Published:** 2023-06-26

**Authors:** Ashley Chory, Emma Gillette, Grant Callen, Juddy Wachira, Nadia A. Sam-Agudu, Keosha Bond, Rachel Vreeman

**Affiliations:** ^1^ Arnhold Institute for Global Health, Icahn School of Medicine at Mount Sinai, New York, NY, United States; ^2^ Center for Global Health, Indiana University School of Medicine - Lafayette, West Lafayette, IN, United States; ^3^ Department of Media Studies, School of Literature, Language and Media, University of the Witwatersrand, Johannesburg, South Africa; ^4^ Department of Mental Health and Behavioral Sciences, Moi University, Eldoret, Kenya; ^5^ School of Medicine, Institute of Human Virology, Baltimore, MD, United States; ^6^ Institute for Human Virology, Institute of Human Virology Nigeria, International Research Centre of Excellence (IRCE), Abuja, Nigeria; ^7^ School of Medical Sciences, University of Cape Coast, Cape-Coast, Ghana; ^8^ Department of Community Health & Social Medicine, The City University of New York, New York, NY, United States; ^9^ Center for Interdisciplinary Research on AIDS Yale University, New Haven, CT, United States

**Keywords:** HIV, gender, knowledge, low and middle-income countries, adolescent

## Abstract

**Objectives:**

This review seeks to critically analyze studies assessing gender differences in HIV-related knowledge among adolescents and young people in low- and middle-income countries.

**Methods:**

Using PRISMA guidelines and searching Pubmed and Scopus online databases, the search strategy combined search keywords with Boolean operators: (HIV OR AIDS) AND (knowledge) AND (gender) AND (adolescents). AC and EG conducted the search and independently reviewed all articles in Covidence software; conflicts were resolved by GC. Articles were included if they evaluated differences in HIV knowledge in at least two groups ages 10–24 and were implemented in a low or middle-income country.

**Results:**

The search resulted in 4,901 articles, of which fifteen studies, implemented in 15 countries, met selection criteria. Twelve evaluated differences in HIV knowledge in school settings; three evaluated participants in clinic settings. Adolescent males consistently scored higher in composite knowledge scores, as well as knowledge of HIV transmission, prevention, attitudes and sexual decision-making.

**Conclusion:**

We found gender-based discrepancies between knowledge, perception of risk and HIV prevalence among youth globally, with boys consistently scoring higher in HIV knowledge. However, there is significant evidence that social and cultural contexts render girls at high risk of HIV infection, and the gaps in girls' knowledge and boys' roles in HIV risk must be addressed urgently. Future research should consider interventions that facilitate discussion and HIV knowledge building across genders.

## Introduction

Globally, there are 1.8 billion adolescents and young people aged 10–24 years, representing one quarter of the world's population, 86% of whom live in low and middle-income countries (LMIC) (1). Adolescence is a critical period of biological, psychological and socioemotional development (2, 3), with new transitions, increasing autonomy and sexual debut for many young adults (4). Sexual debut in adolescence presents new challenges and risks of sexually transmitted infections, and is associated with the highest HIV risk among all age groups (5). Thus, it is critical that HIV education, particularly as related to behavioral risks, sexual health decision-making and HIV prevention strategies are prioritized for this group.

Adolescents and young people living with HIV (AYPLWH) represent the fastest-growing subgroup of people living with HIV; this is due to the increased incidence of new HIV infections within this age group, and the growing number of perinatally-infected youth reaching adolescence and young adulthood (6). As adolescents mature into adulthood, the risk of HIV transmission to sexual partners or infants remains high, necessitating intensive prevention and educational interventions (7). Investigation of adolescent HIV literacy provides an opportunity to assess gaps in knowledge that may perpetuate risk-taking behavior and inform the development of interventions to combat transmission among this high-risk group. Increased HIV-related knowledge has the potential to mitigate risk-taking and other health behaviors that contribute to HIV infection.

Furthermore, investigating potential gender differences in HIV knowledge can provide insight into the need for gender-based interventions to mitigate HIV risk behaviors among adolescents. Harmful gender-based reproductive health norms adopted in adolescence influence long-term health, as they create health inequities for girls and young women, a population most at risk of new HIV infections in high-burden LMICs. The consequences of these harmful norms and inequities include sexual violence, loss of autonomy and agency, and higher rates of sexually transmitted infections including HIV. This warrants a gender-based investigation of factors-including HIV knowledge or lack thereof- associated with high rates of HIV transmission or of strategies and opportunities for prevention.

Investigating differences in HIV literacy across gender allows for better understanding of the relationship between gender and HIV knowledge-related determinants of HIV transmission, especially as it relates to sexual behavior. Better understanding of the relationships between gender, HIV knowledge and HIV transmission can inform the development of responsive interventions to address gender norms that negatively impact HIV knowledge acquisition. This review seeks to critically analyze studies assessing gender differences in HIV-related knowledge among adolescents in LMIC. It aims to identify specific HIV-related topics in which adolescents' knowledge and understanding may differ based on gender, to inform tailored education strategies for the prevention of HIV in this population. The studies included in this review compare outcomes between adolescents and young people assigned male and female at birth and will refer to them as girls and boys throughout; this review reflects sex assigned at birth and not necessarily gender identity.

## Methods

### Search strategy and selection criteria

We used the Preferred Reporting Items for Systematic Reviews (PRISMA) guidelines to conduct this review (8). We searched the PubMed and Scopus online databases for peer reviewed articles and searched the grey literature through the HIV/AIDS Clearing House, USAID Development Experience Clearinghouse, UNESCO HIV and AIDS Education Clearinghouse, and the WHO and UNAIDS websites. The search strategy was completed on 18 August 2021 and combined search keywords with Boolean operators: (*HIV* OR *AIDS*) AND (*knowledge*) AND (*gender*) AND (*adolescents*). The search was run again on 18 October 2022, which returned no additional articles meeting inclusion criteria.

To be included, articles must have met the following criteria: (1) quantitatively evaluated differences in HIV knowledge and perspectives of at least two groups with different sex assigned at birth; (2) included adolescents and young people between ages 10–24 years; (3) included at least one quantitative HIV knowledge outcome measure, and (4) implemented in a LMIC as defined by the World Bank Country Classification (9). There were no exclusion criteria related to study design, provided they met inclusion criteria and were peer-reviewed research. There were no time period limits for this search.

### Data extraction and analysis

**Figure 1:**
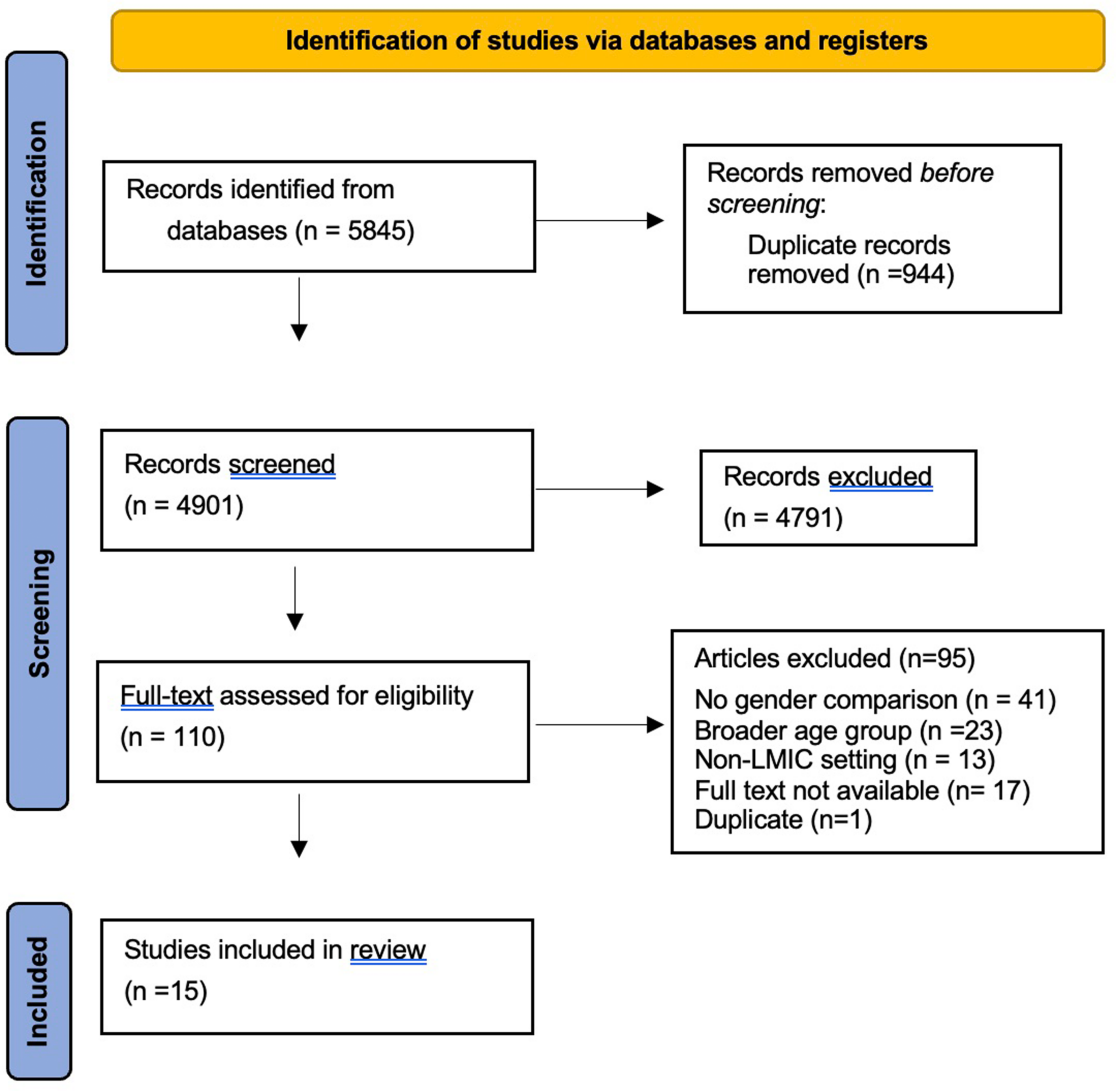
PRISMA diagram 2020 PRISMA diagram.

Two authors (AC and EG) conducted the search in Pubmed and Scopus online databases and exported articles into Covidence software for managing reviews (10). The search strings (*HIV* OR *AIDS*) AND (*knowledge*) AND (*gender*) AND (*adolescents*) were used in both databases, returning a total of 5,485 articles. Covidence software removed duplicates and AC and EG independently reviewed all article titles and abstracts to determine whether the studies met the inclusion criteria. Conflicts were resolved by a third reviewer (GC) to reach consensus. AC and EG reviewed the full text articles to determine if studies should be included, and GC provided secondary review of conflicts. References from included articles were reviewed and screened for additional publications. This process is represented in Figure 1. AC and EG extracted data from the included studies in Covidence for analysis. Article author, journal of publication, publication date, country location of study, region type, study aims and design, instrument(s) used to measure HIV knowledge and topics assessed, target population, sample size and sex distribution of sample, enrollment setting, and results were extracted. A sub-set of the extracted data can be found in Table 1.

**Table 1:** Characteristics of included studies (*N* = 15).

Author, year	Country	Enrollment setting	Study design	Age range	Sample size; male/female	Tool to measure HIV knowledge HIV domains assessed *(overall knowledge, facts and myths, beliefs, behaviors and attitudes)*
Goncalves, 2013 (23)	Brazil	Clinic	Prospective cohort study	11	4,452; 2,192/2,260	Structured questionnaire *Domains:* Overall knowledge
Jahanfar, 2008 (12)	Malaysia	Secondary school	Cross sectional, pre/posttest evaluation	15–19	182; 73/109	Structured questionnaire *Domains:* Overall knowledge, beliefs, behaviors and attitudes
Lal, 2000 (13)	India	College setting	Cross-sectional survey	18–22	625; 164/461	Structured questionnaire *Domains:* Overall knowledge
Mahat, 2006 (14)	Nepal	Secondary School	Cross sectional	13–17	150; 81/69	Structured questionnaire *Domains:* Overall knowledge, facts and myths
Maswanya, 1999 (15)	Tanzania	Secondary school and college	Cross-sectional questionnaire survey	16–24	1,041; 419/622	Questionnaire developed from WHO “Research Package: Knowledge, Attitude, Beliefs and Practices on AIDS (KABP) Phase 1” *Domains:* Overall knowledge, facts and myths, beliefs, behaviors and attitudes
Mwamwenda, 2014 (4)	Kenya	Secondary school	Survey, descriptive	16–18	157; 88/69	Self-developed questionnaire *Domains:* Overall knowledge
Nabunya, 2021 (11)	Uganda	Clinic	Cluster RCT	10–16	702; 306/396	Self-developed interview administered assessment *Domains:* Overall knowledge, beliefs, behaviors and attitudes
Oljira, 2013 (16)	Ethiopia	Secondary school	Cross-sectional school-based survey	14–19	2,766; 1,995/781	Structured questionnaire adapted from WHO sexual and reproductive health questionnaires *Domains:* Overall knowledge, facts and myths
Othman, 2015 (17)	Iraq	Secondary school	Cross-sectional	14–21	437; 221/216	Structured, self-administered questionnaire *Domains:* Overall knowledge
Oyo-Ita, 2005 (18)	Nigeria	Secondary school	Observational	10–22	580; 272/308	Semi-structured questionnaire *Domains:* Overall knowledge
Pinder-Butler, 2013 (19)	Bahamas	Secondary school	Cross-sectional	11–14	354; 195/159	Structured self-administered questionnaire *Domains:* Overall knowledge
Savaser, 2003 (20)	Turkey	Secondary school	Descriptive, correlative study	9th & 11th graders	705; 400/305	Self-developed questionnaire *Domains:* Overall knowledge, beliefs, behaviors and attitudes
Sekera, 2020 (21)	Czech Republic	Secondary school	Cross-sectional	15–19	1,942; 1,073/869	Self-developed questionnaire *Domains:* Overall knowledge, beliefs, behaviors and attitudes
Shamu, 2020 (24)	South Africa	Clinic—national health survey	Baseline cross-sectional data from community HIV prevention intervention	18–24	1,955; 973/982	Adapted from UNAIDS conceptualization of HIV Knowledge tool Domains: Overall knowledge, beliefs, behaviors and attitudes
Zhao, 2010 (22)	China	Secondary school	Survey study	Mean 15.2	995; 495/487	Self-administered questionnaire *Domains*: Overall knowledge, facts and myths

## Results

After conducting searches in all target databases, we identified 5,845 potentially relevant articles and reports (Figure 1). After removing duplicates, a total of 4,901 peer-reviewed articles and gray literature remained for title and abstract review. Full text review was completed for 110 articles, followed by review of references included in reviews and articles meeting inclusion criteria. Ninety-five articles were excluded after full text review because they did not include a gender-specific comparison of HIV knowledge (*n* = 41), did not take place in a LMIC (*n* = 13), included a broader age group (*n* = 23), or full text could not be located (*n* = 17). One duplicate identified through the references of an included article was removed at that stage (11). Data extraction was conducted for 15 peer-reviewed articles.

The 15 studies that met selection criteria varied by study design, enrollment location and outcome measures (Table 1). In total, the included studies enrolled 17,043 adolescents between age 10 and 24 years, comprising 8,947 (52%) males and 8,096 (48%) females. The studies covered four different study designs: randomized control trials, cross-sectional one-time survey assessments, cross-sectional pre-posttest assessments, and prospective cohort studies, and only two studies were interventional (11, 12). Most of the included studies (*N* = 12) evaluated differences in HIV knowledge in the school setting (12–22), particularly among secondary school- and college-aged students; three studies evaluated these differences in the clinic setting (11, 23, 24). Furthermore, most included studies (*N* = 12) utilized study-developed HIV knowledge outcome measures (4, 11, 12, 13, 14, 17–23), while three studies used adapted UNAIDS or WHO tools to evaluate HIV knowledge (15, 16, 24). The selected studies reported a range of outcomes that are presented in Table 2.

**Table 2:** Study aims and statistically significant findings.

Author, year	Study aim	Statistically significant findings
Goncalves, 2013 (23)	Analyze the effect of demographic and socioeconomic factors and sexual education received at school and at home on the level of knowledge about HIV transmission in a samples of adolescents aged 11 years of age living in Southern Brazil	Percentage of error was higher among boys for the following transmission questions: a) Heterosexual intercourse (*p* < 0.001) b) Sharing syringes (*p* < 0.001) c) Hugging someone living with HIV (*p* = 0.018) Regarding HIV infection through homosexual intercourse, the percentage of error in the crude analysis was higher among females (*p* = 0.019)
Jahanfar, 2008 (12)	Investigate the effectiveness of a two hour lecture type sex education program on students’ knowledge and perception towards HIV in Malaysia	• There was an increase in the level of knowledge of both genders after attending the sex education program (25.4–27.7 for males and 24.8–27.3 for females) • Females had a lower mean score of knowledge compared to male for both pre and posttest (24.8 vs. 25.4 and 27.3 vs. 27.7 respectively) • Females reached a higher mean score of perception when compared with male subjects for both pre-test (13.1 vs. 12.5) and posttest (13.2 vs. 12.3)
Lal, 2000 (13)	Study the knowledge and attitude towards HIV, STDs, and sexuality among college students in Kerala; investigate the impact of gender and place of residence on the student's knowledge and attitudes	Males’ overall knowledge score was significantly higher than females (*p* < 0.001) Males had better knowledge of: a) Symptoms of common STDs b) Increased risk of HIV infection associated with acquisition of STDs c) Benefits of total sexual abstinence in the prevention of HIV infection More females believed that HIV was curable and that sexual contact with a familiar person was risk free
Mahat, 2006 (14)	To explore Nepalese adolescents’ knowledge, attitudes and beliefs about HIV and to identify differences in knowledge of HIV by gender	Males had higher: a) Overall knowledge scores (*p* = 0.009) b) Transmission knowledge scores (*p* = 0.034) c) Prevention knowledge scores (*p* = 0.003) d) Perceived level of knowledge (*p* < 0.001)
Maswanya, 1999 (15)	Analyze the relationship between risk behaviors and student's knowledge, attitudes, and risk perception of HIV in Tanzania	Males scored better than females: a) HIV prevention b) Had heard about HIV (OR: 10.14; 95% CI: 6.86–14.99) c) Attitude that friends were at greater risk of HIV than themselves (OR: 1.56; 95% CI: 1.21–2.02)
Mwamwenda, 2014 (4)	Assess HIV knowledge among high school boys and girls in Nairobi, Kenya	*Females performed better than males* a) Transmission mode: sharing a cigarette with a PLWH (*p* < 0.05) b) Transmission mode: sharing of food with a PLWH (*p* < 0.10) c) Transmission mode: Mosquito bites (*p* < 0.001) d) Transmission mode: taking care of a PLWH (*p* < 0.001) e) Cure for HIV (*p* < 0.01) f) Avoidance of those who are infected (*p* < 0.001) g) Children living with HIV should attend school with uninfected children, with more females supporting the statement than males (*p* < 0.01) h) Sharing a bed with a person living with HIV; females expressing that there would be no problem doing so (*p* < 0.001) *Males performed better than females* i) Transmission mode: kissing a PLWH (*p* < 0.00) j) Transmission mode: sharing clothes with a PLWH (*p* < 0.05) k) The chance of contracting HIV was denied by more females than males (*p* < 0.001) l) Attempts to avoid relationships with girls or boys for fear of HIV transmission; males being more careful than females (*p* < 0.10) m) Feel comfortable sitting next to a PLWH (*p* < 0.05)
Nabunya, 2021 (11)	(1) What are the HIV general knowledge, clinical knowledge and prevention attitudes held by AYPLWH in Uganda? (2) Is gender associated with the differences (if any), in HIV general knowledge, clinical knowledge and prevention attitudes among AYPLWH? (3) Does participating in an economic empowerment intervention associated with HIV general knowledge, clinical knowledge, and prevention attitudes among AYPLWH?	*Baseline assessments* a) Males more likely to reject myth that undetectable VL means there is no virus left in the body (*p* = 0.02) b) Females were more likely than boys to agree with the two HIV transmission myths: you can get HIV from a mosquito bite (*p* < 0.01); and from using the same washing basin with an HIV infected person (*p* = 0.02) c) Males more likely to think as a teenager, HIV is a risk to health (*p* = 0.01) d) Males more likely to think you should use a condom even if you know your partner well (*p* = 0.05) *12 Month follow up assessments* *Boys reported higher scores on:* a) HIV general knowledge (<0.01) b) HIV clinical knowledge (<0.01) c) Favorable prevention attitudes (<0.01) d) Males were more likely than females to agree that there is an HIV test (*p* = 0.01), and that anyone can become infected with HIV (*p* = 0.02) Females were more likely than males to reject the myth that using birth control pills can protect a woman from HIV infection (*p* = 0.02)
Oljira, 2013 (16)	Assess the level of comprehensive HIV knowledge and the factors associated with it among in-school adolescents in eastern Ethiopia	• Males were more likely to have comprehensive HIV knowledge than females (*p* = 0.006) • Females were less likely to have comprehensive pregnancy knowledge
Othman, 2015 (17)	Assess the knowledge of high school students about HIV in Erbil city; determine associations between knowledge and socio-demographic characteristics	HIV knowledge was significantly higher among males than females (*p* < 0.001)
Oyo-Ita, 2005 (18)	Assess the impact of media, workshops, peer education and printed materials on HIV awareness in Nigeria	Males were more knowledgeable than women: a) Etiological agent of HIV (*p* = 0.05) b) Sex as a mode of transmission (90.8% vs. 89.3%, *p* = 0.00)
Pinder-Butler, 2013 (19)	Determine gender differences in HIV knowledge and sexual behavior patterns of junior high school students in New Providence, Bahamas	Males were more accurate regarding HIV transmission by mosquito bites than females (*p* = 0.008)
Savaser, 2003 (20)	Determine high school students’ knowledge and attitudes about HIV	Total knowledge scores of males were significantly higher than those of females (*p* < 0.000)
Sekera, 2020 (21)	Assess HIV health literacy among secondary school adolescents	Women showed a higher level of overall HIV knowledge than men (*p* = 0.042)
Shamu, 2020 (24)	Assess the level of and factors associated with HIV knowledge and condom use at last sex among young men and women aged 18–24 years	Males were more knowledgeable than females in: a) Overall HIV knowledge (*p* = 0.027) b) Reduction of risk by having fewer sexual partners (*p* = 0.028) c) HIV cannot be cured (*p* < 0.0001) Being female was associated with lower odds of having high HIV knowledge (AOR: 0.75, 95% CI: 0.58–0.97)
Zhao, 2010 (22)	Examine students’ sources of HIV information, assess overall knowledge and explore gender and grade differences in HIV awareness and knowledge	Males were more knowledgeable than females in: a) Overall knowledge (*p* < 0.01) b) Definition and symptoms (*p* < 0.0001) c) Treatment and prevention (*p* < 0.01) d) False transmission modes (*p* < 0.01) Females were more knowledgeable about true transmission modes (*p* < 0.01)

### Overall HIV knowledge

Eleven of the 15 included studies reported overall HIV knowledge scores. The majority of these 11 studies (*N* = 9) reported significantly higher knowledge among males as compared to females (11–14, 16, 17, 20, 22, 24). These findings were true across several countries; males demonstrated higher overall knowledge assessments in studies conducted in Malaysia, India, Nepal, Uganda, Ethiopia, Iraq, Turkey, South Africa, and China. Six of these nine studies were conducted in secondary schools (12, 14, 16, 17, 20, 22) while the other three were conducted in college (13) and clinic settings (11, 24).

A minority (*N* = 2) of studies reported more varied findings regarding overall HIV knowledge (19, 21). In the Czech Republic, secondary school girls showed a significantly higher level of overall HIV knowledge than boys (*p* = 0.042) (21). Pinder-Butler and colleagues reported no differences in overall HIV knowledge between secondary school boys and girls in the Bahamas (19). Additionally, among secondary school adolescents in Nepal, Mahat and colleagues found that boys scored significantly higher than girls on self-perception of their level of HIV knowledge (*p* < 0.001) (14). Lastly, Nabunya and colleagues reported no gender differences in knowledge among adolescent clinic patients in Uganda regarding a cure for HIV (11).

### HIV knowledge by gender before and/or after an intervention

#### Nabunya et al, Uganda

In Uganda, Nabunya and colleagues investigated HIV clinical knowledge among 702 adolescents (306 males vs. 396 females) before and one year after an economic empowerment intervention to improve ART adherence (11). At baseline, males were more likely to reject the myth that undetectable viral load meant that there is no virus left in the body (*p* = 0.02). At follow up, males were more likely to agree that: (1) there is an HIV test (*p* = 0.01) and (2) that anyone can become infected with HIV (*p* = 0.02). Males were also more likely to know that: (1) CD4 count measures how many “soldier” cells are in the body to fight HIV (*p* < 0.01); (2) low CD4 count means fewer “soldier” cells to fight infections (*p* < 0.01); (3) viral load tests measure how much HIV is in the blood (*p* < 0.01); (4) resistant virus means that medicine no longer works to lower or slow down the virus (*p* < 0.01); and (5) the virus can be resistant if medication doses are missed (*p* < 0.01) In addition, males were more likely to reject the following myths: (1) it's ok for a person to stop taking their medication if their CD4 count is high or if they feel healthy (*p* < 0.01) and; (2) undetectable viral load means there is no virus left in the body (*p* = 0.01). Nabunya and colleagues hypothesized that the observed differences may be rooted in gender and cultural norms in which girls and women have more restricted opportunities for education and are expected to know less about sex, boys and men have decision-making power around safe sex, and open discussions around sex among adolescents is considered taboo (11).

#### Jahanfar et al, Malaysia

In Malaysia, Jahanfar and colleagues investigated HIV knowledge among 182 adolescents (73 males vs. 109 females) before and immediately after a two-hour talk on sexual education (12). The authors reported an increase in HIV knowledge among both males and females after the sex education program; mean scored increased from 25.4 to 27.7 for males and from 24.8 to 27.3 for females. As compared to their male counterparts, females had a lower mean score of knowledge for both pre and posttest assessments (pre-test scores: 24.8 female vs. 25.4 male and posttest scores: 27.3 female vs. 27.7 male).

### HIV transmission and prevention: facts vs. myths

Eight included studies assessed knowledge by asking participants to identify facts vs. myths of HIV transmission modes (4, 11, 14, 18–20, 22, 23). In Nepal, Mahat and colleagues reported that males were more knowledgeable than females and had higher overall transmission knowledge scores (*p* = 0.034), particularly in HIV prevention (*p* = 0.003) (14). In Nigeria, Oyo-Ita and colleagues reported no gender differences in overall knowledge regarding modes of HIV transmission (18). In Turkey, Savaser reported no gender differences in knowledge about risk categories for individuals or key groups (20).

#### Myths

In China, Zhao et al. included questions regarding both true and false transmission modes; males were more knowledgeable in identifying false transmission modes (*p* < 0.01); females were more knowledgeable about true transmission modes (*p* < 0.01) (22). Lal and colleagues reported that both males and females were equally misinformed about the risk of getting HIV by donating blood to a blood bank (13). There were no gender differences in the identification of the following myths as modes of HIV transmission: use of a common toilet seat, and shaking hands with a PLWH (4). The belief that HIV is punishment for engaging in sex out of wedlock was rejected equally by both sexes (4).

Three studies (in Kenya, Uganda and the Bahamas) reported that males more accurately identified HIV-related transmission and prevention myths. Nabunya and colleagues found that males were less likely to agree with the myth that HIV can be transmitted from a mosquito bite (*p* < 0.01), or from using the same washing basin as a PLWH (11). Pinder-Butler found that males had more accurate information regarding HIV transmission by mosquito bites than females (*p* = 0.008) (19). In Kenya, sharing clothes with a person living with HIV was rejected as a mode of transmission by more males than females (*p* < 0.05) (4).

Three included studies reported that females were able to correctly identify HIV-related transmission and prevention myths. Mwamenda and colleagues report that more Kenyan females rejected the myth that mosquito bites led to HIV transmission (*p* < 0.001); taking care of a PLWH (*p* < 0.001), sharing food (*p* < 0.10) and sharing a cigarette (*p* < 0.05) were also rejected as modes of transmission by more females than males (*p* < 0.001) (4). Sharing syringes was discussed in one study, where the percentage of error was also lower among females (*p* < 0.001) (23). Percentage of error was lower among females for hugging a PLWH as a transmission mode (*p* = 0.018) (23). Nabunya and colleagues investigated HIV prevention knowledge among adolescent males and females in Uganda; females were more likely than males to reject the myth that using birth control pills can protect a woman from HIV infection (*p* = 0.02) (11).

#### Facts

Several studies reported results for individual questions regarding sexual intercourse as a mode of transmission (4, 11). One study reported no differences in identifying sex with a PLWH as a mode of transmission (4). One study included questions regarding HIV transmission from mother-to-child and found no gender differences in knowledge (11). Four studies (in Nigeria, Uganda, Kenya and Brazil) found that males had more accurate knowledge of true transmission modes and prevention strategies. Oyo-Ita and colleagues reported that Nigerian males were more knowledgeable than females in identifying sex as a mode of transmission (*p* = 0.00) (18). In Uganda, males were more likely to report accurate prevention attitudes, for example, acknowledgement that HIV impacts health (*p* = 0.01), and that it is appropriate to use a condom even with a known partner (*p* = 0.05) (11). Goncalves et al. reported a lower percentage of error among Brazilian males in identifying homosexual intercourse as an HIV transmission mode (*p* = 0.019) (23). Only one study, from Brazil, reported higher scores among females related to accurate identification of HIV- transmission modes (23). Goncalves and colleagues reported a lower percentage of error among females in identifying heterosexual intercourse as a mode of HIV-transmission (*p* < 0.001) (23).

#### Behaviors, beliefs and attitudes

Outcomes related to participant beliefs and attitudes were included as an exploratory measure, as beliefs and attitudes related to HIV are influenced by overall knowledge. Five of the included studies evaluated participants' beliefs, behaviors and attitudes regarding HIV. Mwamwenda and colleagues reported that females surveyed in Kenya more commonly believed that (presumably unsafe) sexual relationships with PLWH should be avoided (*p* < 0.001), and believed themselves to have less of a chance of contracting HIV than males (*p* < 0.001) (4). However, males more commonly reported taking action to avoid relationships for fear of HIV transmission (*p* < 0.10) (4). More females supported the idea that children living with HIV should attend school with children without HIV (*p* < 0.01) (4). More females reported no problems sharing a bed with a PLWH (*p* < 0.001); interestingly, more males reported feeling comfortable sitting next to a PLWH (*p* < 0.05) (4).

Nabunya and colleagues found that Ugandan males were more likely to think one should use a condom even if you know your partner well (*p* = 0.05) (11). In contrast, Lal and colleagues reported that more Indian females believed that sexual contact with a familiar person was risk-free, and that banning commercial sex work could control the spread of HIV; however, no statistical tests were conducted to estimate this difference (13). Maswanya and colleagues reported that Tanzanian males more commonly believed that their friends were at greater risk of HIV than themselves (OR: 1.56; 95% CI: 1.21–2.02) (25).

## Discussion

This review identified 15 articles on gender differences in HIV knowledge among adolescents and young people in LMIC. Articles included in this review discuss adolescent knowledge between the ages of 11 and 24, a large age range that represents significant growth and development. Importantly, HIV-related knowledge may evolve as the individual progresses through the adolescent and young adult lifespan. We reviewed findings with respect to overall knowledge, facts vs. myths, and beliefs, behaviors and attitudes. We found that males were consistently found to be more knowledgeable about HIV than females when evaluated through a composite overall score (Table 3). When topics were broken down by question or literacy topic, there was variation in the findings, making it challenging to draw topic-specific conclusions. These findings highlight a critical need for HIV education programs for adolescents to assess knowledge not only broadly but by specific key topics. These key topics include myths and facts about HIV transmission and prevention and sexual decision-making, in which males more commonly reported higher knowledge.

**Table 3:** Summary of HIV literacy results by gender (*N* = 15 studies).

	Boys performed better	Girls performed better	Recommended action(s)
Overall knowledge (*n* = 10)	Nine studies (4, 10–13, 16, 19, 21, 23)	One study (20)	Targeted educational interventions to promote overall HIV literacy among adolescent females.
Facts (*n* = 4)	Four studies (17, 10, 22, Brazil)	No studies	Targeted education interventions on HIV transmission and prevention facts for adolescent females.
Myths (*n* = 5)	Four studies (21, 10, 18, 15)	One study (22)	Targeted education interventions to dispel HIV transmission and prevention myths for adolescent females.
Beliefs, Attitudes, Behaviors (*n* = 5)	Three studies (10, 15, 12)	Two studies (13, 15)	Further exploration of the differences in HIV related beliefs, attitudes and behaviors between adolescent males and females.

Regarding attitudes and behaviors related to HIV, there were concerning findings among females (4, 11, 13, 25). Females were less likely to perceive themselves at risk of contracting HIV, were less careful in avoiding unsafe sex with PLWH, were more likely to believe that sexual contact with a familiar person was low risk (13) and had poorer prevention attitudes regarding condom use (15). For adolescent girls, these findings represent a discrepancy between risk perception and HIV prevalence, as women and girls are much often at higher risk of acquiring HIV than their male counterparts (26, 27). Besides the female physiological vulnerability to sexually transmitted infections (28, 29), higher HIV risk and exposure among women and girls are influenced by gender norms that place them at increased risk. These norms include less access to formal and informal education (including HIV education) (27, 30, 31), early marriage (32, 33) and transactional and intergenerational sex (34–38).

Furthermore, in places where the predominant and most accessible prevention tool is the male condom, girls have limited ability to protect their sexual health, particularly in communities in which males hold more decision-making power regarding condom use. Use and adherence to biomedical HIV prevention strategies, like pre-exposure prophylaxis (PrEP) remains low in LMIC, often because of cost, access, and HIV-related stigma (39). Our findings and those of prior studies support a feminist approach to HIV prevention and relevant education for female adolescents, given their gender-based social vulnerabilities to HIV infection.

Our review identified two interventional studies that reported improvements in general knowledge, clinical knowledge and prevention attitudes regarding HIV. Both studies reported less of an interventional impact among girls (11, 12). These data are important as previous studies have demonstrated that correct knowledge regarding HIV is a powerful mechanism in the promotion of positive attitudes and engagement in safe-sexual practices; programs have focused on transmission knowledge as a means of counteracting misconceptions that lead to risk-taking behavior (40–42).

### Limitations

Our study had some limitations. First, this review was limited to findings from only 15 studies in LMICs, which limits its generalizability to all LMICs. However, it highlights gaps in the geographical scope of research on gender-based evaluation of HIV knowledge among young people in the most high-burden settings. Furthermore, it identifies the knowledge areas of most concern in HIV knowledge by gender, as reported from currently available research. Second, the studies included were quite heterogeneous in design, and did not allow for a more uniform means of analysis. However, we were able to categorize all study findings across three major cross-cutting domains (Overall Knowledge; Myths and Facts; and Beliefs, Behaviors, and Attitudes). Third, across included studies, there were no in-depth assessments of HIV knowledge that took into account the level of formal education for individuals or for male vs. female sub-groups. Fourth, none of the studies meeting inclusion criteria evaluated HIV-related knowledge among sexual and gender minority adolescent groups—who are also highly vulnerable to HIV infection, and experience intense stigma and discrimination. Finally, the studies discussed include adolescents from 11 to 24 years, a broad age range with significant variation in growth and development. Future analyses should consider the differences in HIV knowledge within shorter developmental periods.

## Conclusion

This majority of studies included in this review found that males are generally more knowledgeable about HIV than females, particularly regarding transmission and prevention. There was significant variation in topic-specific findings, so further research is needed to better understand topic-specific nuances in HIV-related knowledge, and gender norms that perpetuate gender-based gaps in HIV knowledge and decision-making power. For HIV prevention to be successful, LMICs with a high burden of HIV should integrate comprehensive HIV education into schools, clinics and other settings in which adolescents spend a significant amount of time. While there is significant evidence on the social and cultural contexts that lead girls and young women to be at particularly high risk of HIV infection, there remains a gap in knowledge of the roles boys and young men play in this continuum. Future research should consider interventions that facilitate discussion between adolescent boys and girls and how to address these gender dynamics effectively.
